# Targeting MUC4 in pancreatic cancer: miRNAs

**DOI:** 10.18632/oncoscience.249

**Published:** 2015-09-12

**Authors:** Nicolas Jonckheere, Fatima Lahdaoui, Isabelle Van Seuningen

**Affiliations:** Inserm, UMR-S1172, Jean-Pierre Aubert Research Center, Team “Mucins, epithelial differentiation and carcinogenesis”, Lille cedex, France; Université Lille 2 Droit et Santé, Lille cedex, France

**Keywords:** MUC4, miRNA, pancreatic cancer

MUC4 is a type I membrane-bound mucin expressed at the apical pole of normal polarized epithelial cells. MUC4 apomucin is characterized by a long hyperglycosylated extracellular domain, Epidermal Growth Factor (EGF)-like domains, a hydrophobic transmembrane domain, and a short cytoplasmic tail. MUC4 also contains NIDO, AMOP and vWF-D domains that are unique in the apomucin family. In cancers, MUC4 and the oncogenic receptor ErbB2 interact physically via the EGF-like domains [1]. MUC4 plays major roles in the behavior of epithelial tumor cells as it promotes proliferation, motility, invasiveness, Epithelial-Mesenchymal Transition (EMT), chemoresistance and tumor growth [1–3]. Pancreatic cancer has been the favored model to decipher the cellular mechanisms and the intracellular signaling pathways associated with MUC4 altered expression.

Pancreatic Ductal Adenocarcinoma (PDAC) is the 4th leading cause of death by cancer worldwide. Its poor survival rate at 5 years (3–5%) and median survival curve (6 months) are the consequences of a late detection and a lack of efficient therapies [4]. Understanding the regulation of early deregulated genes (such as MUC4) will open new avenues in developing tools to target early steps of this deadly cancer. Indeed, MUC4, which is not expressed in healthy pancreas, is neoexpressed as early as PanIN-1A preneoplastic stage. MUC4 overexpression is then sustained toward adenocarcinoma. The prevalence of MUC4 apomucin expression, one of the most differentially expressed genes in PDAC, reaches 83 to 89%.

**Figure 1:**
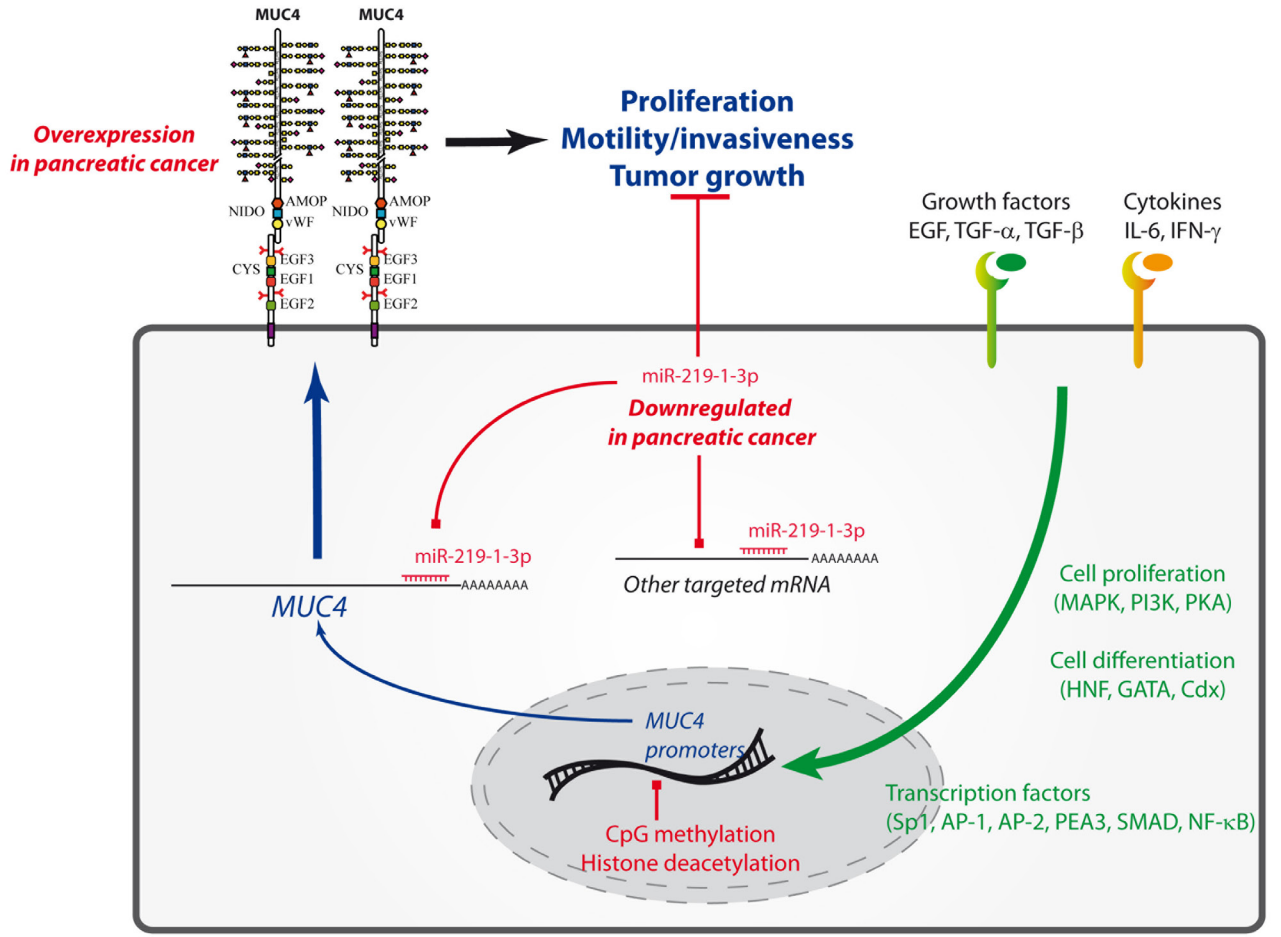
MUC4 transcriptional and epigenetic regulation in pancreatic ductal adenocarcinoma Implication of miR-219-1-3p.

*MUC4* transcription is complex, tightly regulated and involves many signaling pathways (Figure 1). This redundancy and complexity increase the difficulty to efficiently target *MUC4* expression in PDAC. *MUC4* 5′-flanking region contains two active promoters: A TATA-less proximal promoter mainly composed of GC-rich domains and a great density of binding sites for factors known to initiate transcription in TATA-less promoters (Sp1, CACCC box, glucocorticoid receptor element, AP-1, polyomavirus Enhancer Activator-3 (PEA3) and Med-1) and a distal promoter characterized by a TATA box and containing numerous binding sites for both ubiquitous and specific transcription factors (Sp1, AP-1, AP-4, GATA, CREB) [5]. *MUC4* regulation is highly complex and involves a wide range of specific factors such as AP-2, PEA3, IFN-γ and IL6 inflammatory pathways (via STAT1) and CDX-1/-2, HNF-1α/-1β, FOXA1/A2, HNF-4α/-4γ, and GATA-4/-5/-6 endodermal transcription factors [5]. TGF-β is also a strong inducer of MUC4 expression via Smad4 dependent (canonic pathway) and independent (non canonic) pathways (MAPK, PI3K and PKA). Recently, we also showed that MUC4 is a target of K-ras^G12D^ mutation and downstream signaling via both transcriptional and post-transcriptional mechanisms (unpublished). In healthy pancreas, MUC4 expression is repressed by epigenetic mechanisms and heavy methylation of the CpG islands present in its promoters [5]. This hypermethylation by DNMT3A/3B is linked with a repressive histone code including histone deacetylation by HDAC3.

Post-transcriptional regulation by microRNAs (miRNA) is a new promising strategy to control gene expression in cancers that may lead to emerging circulating biomarkers and therapeutic targets [6]. We recently identified miR-219-1-3p as a new negative regulator of MUC4 mucin expression in pancreatic cancer (PC) cells and showed a converse correlation in human pancreatic adenocarcinomatous tissues and in the early steps of pancreatic carcinogenesis (PanINs) in the preclinical Pdx1-Cre; LstopL-Kras^G12D^ transgenic mouse model [7]. We also demonstrated that miR-219-1-3p possesses tumor-suppressive activity as its overexpression leads to reduced proliferation and migrating properties of PC cells via a decrease of cyclin D1 expression and decreased Erk and Akt activation. Intratumoral injection of miR-219-1-3p inhibits pancreatic tumor progression in subcutaneous xenografts highlighting its potential as a therapeutic tool. We believe that the early miR-219-1-3p repression and the resulting increased expression of the transmembrane mucin MUC4 may represent two key events that favor pancreatic tumor progression. MiRNA profiling in non-microdissected human tissues confirmed that among the miRNAs that are downregulated in PDAC compared with normal tissues, miR-219-1-3p emerged as one of the most relevant [8].

Based on our recent work, we propose miR-219-1-3p as a good tumor-suppressor candidate to inhibit MUC4 expression, MUC4-mediated downstream signaling pathways, and MUC4-independent cellular tumor suppressor mechanisms highlighting the therapeutic potential of this miRNA in pancreatic cancer (Figure 1). MiRNA-based therapies are proposed to have the potential to overcome the limitations of current cancer therapies and consequently tumor resistance [3]. Further works are mandatory in order to reach clinical trial.
